# Microglial Activation of GLP-1R Signaling in Neuropathic Pain Promotes Gene Expression Adaption Involved in Inflammatory Responses

**DOI:** 10.1155/2021/9923537

**Published:** 2021-08-31

**Authors:** Le Ma, Peijun Ju, Wei Wang, Jinbao Wei, Weidi Wang, Mengjing Zhao, Khalil Ali Ahmad, Yongxiang Wang, Jinghong Chen

**Affiliations:** ^1^Shanghai Key Laboratory of Psychotic Disorders, Shanghai Mental Health Center, Shanghai Jiao Tong University, School of Medicine, School of Pharmacy, Shanghai 200240, China; ^2^King's Lab, Shanghai Jiao Tong University School of Pharmacy, Shanghai 200240, China

## Abstract

**Background:**

Neuropathic pain is a common chronic pain, which is related to hypersensitivity to stimulus and greatly affects the quality of life of patients. Maladaptive gene changes and molecular signaling underlie the sensitization of nociceptive pathways. We previously found that the activation of microglial glucagon-like peptide 1 receptor (GLP-1R) could potently relieve formalin-, bone cancer-, peripheral nerve injury-, and diabetes-induced pain hypersensitivity. So far, little is known about how the gene profile changes upon the activation of GLP-1R signaling in the pathophysiology of neuropathic pain.

**Methods:**

Spinal nerve ligation (SNL) was performed to induce neuropathic pain in rats. Mechanical allodynia was assessed using von Frey filaments. The expression of IL-10, *β*-endorphin, and *μ*-opioid receptor (MOR) was examined by real-time quantitative polymerase chain reaction (qPCR) and whole-cell recording. Measurements of cellular excitability of the substantia gelatinosa (SG) neurons by whole-cell recording were carried out. R packages of differential gene expression analysis based on the negative binomial distribution (DESeq2) and weighted correlation network analysis (WGCNA) were used to analyze differential gene expression and the correlated modules among GLP-1R clusters in neuropathic pain.

**Results:**

The GLP-1R agonist, exenatide, has an antiallodynic effect on neuropathic pain, which could be reversed by intrathecal injections of the microglial inhibitor minocycline. Furthermore, differential gene expression analysis (WGCNA) indicated that intrathecal injections of exenatide could reverse the abnormal expression of 591 genes in the spinal dorsal horn induced by nerve injury. WGCNA revealed 58 modules with a close relationship between the microglial GLP-1R pathway and features of nerve injuries, including pain, ligation, paw withdrawal latency (PWL), and anxiety. The brown module was identified as the highest correlated module, and the Kyoto Encyclopedia of Genes and Genomes (KEGG) analysis indicated that inflammatory responses were most correlated with PWL. To further unravel the changes of hyperalgesia-related neuronal electrophysiological activity mediated by microglia GLP-1 receptors, whole-cell recording identified that MOR agonism stimulated a robust outward current in the sham groups compared with the spinal nerve ligation (SNL) groups. This inhibitory effect on the SNL group was more sensitive than that of the sham group after bath application of *β*-endorphin.

**Conclusions:**

Our results further confirmed that the GLP-1R pathway is involved in alleviating pain hypersensitivity mediated by spinal microglia activation, and inflammatory responses were the most correlated pathway associated with PWL changes in response to exenatide treatment. We found that the identification of gene regulation in response to GLP-1R activation is an effective strategy for identifying new therapeutic targets for neuropathic pain. Investigation for the activation of spinal microglial GLP-1R which might ameliorate inflammatory responses through gene expression and structural changes is providing a potential biomarker in pain management.

## 1. Introduction

Unbalanced inflammation in the nervous system may contribute to the initiation and maintenance of persistent pain [1]. Cytokines, chemokines, etc., could be released during an inflammatory response and promote maladaptive synaptic plasticity [2, 3]. Additionally, the inflammatory mediators would directly or indirectly regulate the release of neurotransmitters such as glutamate, *γ*-aminobutyric acid, and glycine as well as postsynaptic transmitters, followed by long-term deleterious plastic changes of nerve activity [4]. Neuroinflammation facilitates pain signal transduction and the reconstruction of the nerve loop in the central nervous system (CNS). Furthermore, imbalanced excitability and inhibitory neurotransmission lead to hyperalgesia. Therefore, changes in inflammation and neurotransmission are closely related to the occurrence and maintenance of neuropathic pain. Targeting excessive inflammation could be seen as a therapy for neuropathic pain.

Microglia are the major source of multiple inflammatory cytokines in CNS, and increasing evidence inferred that microglia could drive central sensitization and pain hypersensitivity via a microglial mediator [5]. However, direct targeting of microglial activation and neuroinflammation via inhibition of microglia may produce side effects such as infection and inflammation [6]. Strategies that could control abnormal microglial activation and excessive neuroinflammation as well as promote a return to neural homeostasis for relieving pain should be considered.

Glucagon-like peptide 1 receptor (GLP-1R) signaling, which is specifically expressed in the microglia of the spinal dorsal horn, was found to be involved in a number of biological processes, including inflammation, neuroprotection, and synaptic plasticity [7–10]. Cumulative studies from our laboratory illustrated that GLP-1R agonism could effectively relieve pain hypersensitivity through increasing the biomarkers of M2 in microglia associated with expression of IL-10, CD206, IL-4, and Arg1 [10–13]. Specifically, intrathecal injection of exenatide completely abolished the pain states of bone cancer-, peripheral nerve injury-, formalin-, and diabetes-induced hypersensitivity through IL-10 and *β*-endorphin expression [11]. Furthermore, bath application of GLP-1R agonism abolished spinal glutamatergic transmission, and those inhibitory effects could be reversed by microglial inhibitor minocycline [13]. Moreover, exenatide showed antiallodynic effects by stimulating cAMP, p-protein kinase A (PKA), p-p38, and p-cyclic adenosine monophosphate (cAMP) response element binding protein (CREB) in a dose-dependent manner. Genetical knockdown of p38*α* or inhibition of PKA suppressed exenatide-induced p38/*β*-endorphin expression [14]. Morroniside, which was extracted from traditional medicinal herbs, was identified as the agonist of GLP-1R and could suppress peripheral nerve injury-induced hypersensitivity mediated by microglial autocrine IL-10 and *β*-endorphin expression [8, 12, 15]. These data collectively demonstrate that the antinociceptive pathway of GLP-1 in the microglia plays a crucial role in the pathophysiological condition of neuropathic pain in the CNS. However, the large scale of mechanisms linking GLP-1R in neuropathic pain remains largely unraveled. Untargeted approaches may be required to highlight GLP-1R agonist-mediated differences in global signaling networks.

In our study, we aim to determine the gene expression profiles that contributed to neuropathic pain before and after activation of the spinal dorsal horn microglial GLP-1R signaling by RNA sequencing. We found that the microglial GLP-1R agonist, exenatide, rescued the expression of 591 genes in the dorsal spinal cord with spinal nerve injury, and WGCNA identifies critical brown modules correlating with the GLP-1R pathway in neuropathic pain. Our results further illustrated that GLP-1R-induced antiallodynia was correlated with inflammatory responses in neuropathic pain. Furthermore, the GLP-1R pathway was critically involved in spinal synaptic plasticity in pain as verified with electrophysiological recordings. The application of MOR agonists showing inhibitory effect on neuronal excitability was less likely abolished in rats with neuropathic pain, suggesting the involvement of GLP-1R/IL-10/*β*-endorphin in the neuronal plasticity of pain. Taken together, our study reveals that GLP-1R signaling, stimulating IL-10/*β*-endorphin expression, has potential to inhibit the excitability of lamina II neurons of the substantia gelatinosa (SG) after pharmacological blocking of the MOR.

## 2. Materials and Methods

### 2.1. Chemicals and Reagents

Exenatide was synthesized from Dan Gang Peptides Co. (Hangzhou, China); [D-Ala2, N-Me-Phe4, Gly5-ol]-enkephalin (DAMGO) was obtained from Phoenix (Burlingame, CA, USA), while minocycline was purchased from Yuanye Biotech (Shanghai, China). D-Phe-Cys-Tyr-D-Trp-Arg-Thr-Pen-Thr-NH2 (CTAP) were purchased from Abcam.

### 2.2. Animals

Male Wistar rats were raised in a controlled temperature (23 ± 1°C) and humid environment on a 12 h light/dark cycle with lights on at 7:00 AM. Food and water were provided *ad libitum*. All experiments were performed in accordance with the Animal Care and Welfare Committee of Shanghai Jiao Tong University and followed the animal care guidelines of the National Institutes of Health.

### 2.3. Spinal Nerve Ligation and Behavioural Testing

Rats (200-250 g) were anaesthetized by intraperitoneal injection of pentobarbital sodium (50 mg/kg), the left L5 paralleled to the spinal cord was gently dissected with a man-made glass hook, and the nerves were gently ligated with 6-0 silk sutures. The L6 nerve, vertical to the spinal cord and looking more thinner than L5, was operated identically. The sham rats were operated identically without ligations of L5 and L6. After those procedures, the wound was sutured before giving broad antibiotics preventing infections. The rats were allowed to recover for 14 days, and the spinal nerve ligation (SNL) rats developed significant hypersensitivity to mechanical stimuli on the operated side with paw withdrawal thresholds < 8 g. No motor impairments were excluded from this study.

For mechanical threshold testing, all rats were handled by the investigators for 4 days [16]. The rats were randomly divided into groups and blinded to the investigators. The rats were individually placed in a plastic box and acclimated for 30 min before the tests. Mechanical threshold testing was performed with 2290 CE electrical von Frey hair (IITC Life Science, Woodland Hills, CA, USA) ranging from 0.1 to 90 g. The upward force stimulated suddenly withdrew from operated hind paws including withdrew, lifting, and licking, while the lowest force was recorded as the threshold. The procedure was repeated three times at 1 min intervals. The average of the recorded thresholds was determined as the mechanical threshold.

### 2.4. Intrathecal Catheterization and Injection in Rats

Before ligation of L5 and L6, a catheter (0.28 nm inner diameter and 0.61 nm outer diameter, PE-10, Clay Adams, Parsippany, NJ, USA) was placed in the lumbar spine of the rats, and the typical whipping was observed under inhaled isoflurane anaesthesia. Two joints in the catheter were used tightly located near the ilium and neck to prevent displacement. The following day, intrathecal injection of 10 *μ*L of 4% lidocaine in artificial cerebrospinal fluid (ACSF: 125 mM NaCl, 3 mM KCl, 1.25 mM NaH_2_PO_4_, 26 mM NaHCO_3_, 1 mM MgCl_2_, 2 mM CaCl_2_, and 10 mM D-glucose, pH 7.3) was administered, followed by bilateral paralysis and claudication, while the other rats were excluded from this study. For intrathecal delivery, 10 *μ*L of the drug solution was administered through a 50 *μ*L microinjector followed by a 15 *μ*L artificial cerebrospinal fluid (ACSF) flush. In our study, 100 *μ*g minocycline and 100 ng exenatide were intrathecally injected.

### 2.5. RNA Isolation and Quantitative Reverse Transcription PCR (qRT-PCR)

The ipsilateral spinal enlargements L3-L5 of the sham and SNL groups from 4 rats in each group were gently isolated into cold ACSF. Total RNA was extracted using TRIzol, and the ratio of 260/280 > 1.8 was chosen for further experiments. Using the First Strand cDNA Synthesis Kit (Thermal, K1622), 1 *μ*g mRNA was used for cDNA synthesis. The primers used were as follows: proopiomelanocortin (POMC) (exon 2–3) forward primer: CCTATCGGGTGGAGCACTTC and reverse primer: TGGCTCTTCTCGGAGGTCAT, IL-10 forward primer: GGCTCAGCA CTGCTATGTTGCC and reverse primer: AGCATGTGGGTCTGGCTGACTG, and GAPDH forward primer: CCAAGGTCATCCATGACGAC and TCCACAGTCTTCTGAGTGGC. All primers were manufactured according to a previous study [11]. Real-time quantitative PCR was performed using Roche SYBR qPCR Mix and identified to be specific as assessed by a melting curve. The relative expression of IL-10 and POMC was calculated using the 2^-*ΔΔ*CT^ method after normalization to GAPDH.

### 2.6. Spinal Slice Preparation

The L3-L5 spinal cord was quickly separated under inhaled isoflurane anaesthesia and transferred into ice-cold high-sucrose ACSF containing 234 mM sucrose, 3.6 mM KCl, 1.2 mM MgCl_2_, 1.2 mM NaH_2_PO_4_, 12 mM glucose, 2.5 mM CaCl_2_, and 25 mM NaHCO_3_ for 90 s. The spinal slices were cut using a Leica VT-1200S vibratome (Wetzlar, Germany), the velocity was 0.06 mm/s, and the amplitude was 1.00 mm. The 400 *μ*m slices were transferred to 32°C oxygenated ACSF for 30 min and cooled to room temperature.

### 2.7. Whole-Cell Recordings

Whole-cell recording was conducted in the ipsilateral dorsal horn neurons of the substantia gelatinosa with a 4-5 M*Ω* pipette (1.0 mm outer diameter, 0.5 mm inner diameter; Sutter Instruments, Novato, CA, USA). The excitability of spinal neurons from lamina II was recorded before and after bath application of 1 *μ*mol *β*-endorphin or 1 *μ*mol DAMGO using pipettes containing an internal solution (135 mM K-gluconate, 0.5 mM CaCl_2_, 2 mM MgCl_2_, 5 mM EGTA, 5 mM HEPES, and 5 mM Mg-ATP, pH 7.3) under current clamp conditions. All the neurons were clamped at −70 mV, and current injections were given with step intervals of 20 pA from -80 to 400 pA over a period of 500 ms. The spike numbers were calculated as the excitability according to the currents injected into the neurons by Clampfit 10.7.

### 2.8. RNA Extraction and Sequencing

The spinal dorsal horn was rapidly dissected on ice from the fresh spinal cord. Total RNA was extracted from 11 samples using the RNeasy Mini kit (Qiagen) for further library preparation and sequencing by Illumina NovaSeq 6000 PE150. Bioinformatic analyses of mRNA sequencing raw reads were performed by FASTQCT. The reads were mapped to the ensemble database of rat norvegicus (Rat (Rnor_6.0)). Normalized gene counts for identifying distinct genes affected by different treatments were chosen for bioinformatic analyses. Differential expression analyses were conducted using R package DESeq2 (log_2_ (fold change) > log_2_ (1.5) and *p*adj < 0.05).

### 2.9. Weighted Gene Coexpression Network Analysis (WGCNA)

The coexpression network was analyzed using weighted gene coexpression network analysis (WGCNA), reshape2, and stringr packages in R version 4.0.4 (2021-02-15). The coexpression network was calculated by transforming the gene coexpression correlation matrix into the adjacency matrix and subsequently transferring it into the topological overlap matrix (TOM). When TOM = 0, there is no correlation between gene expression and other gene expressions. Based on the principle of data dissimilarity, WGCNA further polished TOM data and divided gene expression into gene modules. The cluster dendrogram was obtained by merging highly similar data sets. Highly similar expression relationships were grouped and divided into modules. Each module was identified and correlated with behavioural phenotypes such as ligation, nerve injury, pain, anxiety, and paw withdrawal latency.

### 2.10. Data Evaluation and Statistics

GraphPad Prism 7.0 (GraphPad Software) was used for data analysis. The data were analyzed using two independent sample Student's *t*-test and one-way or repeated-measures two-way ANOVA, followed by Tukey's post hoc tests for multiple comparisons. Data are summarized as the mean SEM. Statistical significance was set at *p* < 0.05. All data were processed using CorelDraw 2019.

## 3. Results

### 3.1. Intrathecal Delivery of Exenatide Inhibited Mechanical Allodynia in Neuropathic Pain via Microglial GLP-1R Activation

**Figure 1:**
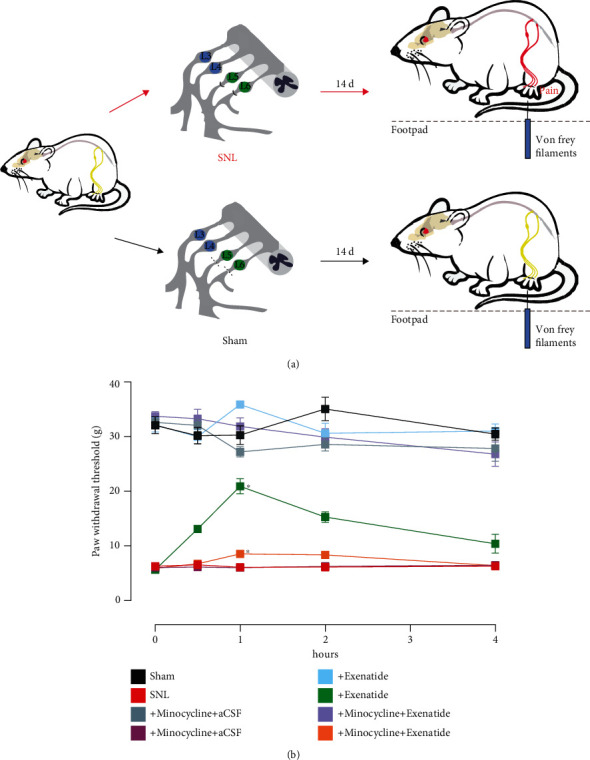
Schematic diagram of the rat operation and behavioural testing (a). Effects of glucagon-like peptide 1 receptor agonist exenatide in the activation of microglia in SNL-induced rats (b). Rats with spinal nerve ligation-induced neuropathy, received intrathecal injections of vehicle, exenatide (100 ng), or pretreatment of minocycline (100 *μ*g) 4 h. Data are presented as means ± SEM (*N* = 5 per group). ^∗^*p* < 0.05, analyzed by the repeated measures two-way ANOVA followed by the Tukey post hoc test.

The analgesic effects of the GLP-1R agonist exenatide were assessed in rats with spinal nerve ligation-induced neuropathic pain, and sham rats were identically operated on without ligation (Figure 1(a)). The rats from the SNL and sham groups were individually administered vehicle (10 *μ*L), exenatide (100 ng), or the microglial metabolic inhibitor minocycline (100 *μ*g). Mechanical thresholds were tested with noninvasive monofilament stimulus on operated hind paws before and at 0.5, 1, 2, and 4 h. Intrathecal injection of exenatide significantly suppressed mechanical allodynia after 1 h of incubation, without affecting the basal thresholds in the sham group. Pretreatment with minocycline (100 *μ*g) dramatically inhibited microglial functions in neuropathic pain and exhibited completely abolished exenatide-induced analgesic effects 1 h later. In the sham group, exenatide or minocycline did not affect the basal thresholds of the sham groups at 4 h (Figure 1(b)). These results confirmed that activation of microglia GLP-1R is specifically effective in alleviating pain hypersensitivity states.

### 3.2. Microglial GLP-1R Agonist Exenatide Ameliorated Abnormal Cellular Signaling in Neuropathic Pain

**Figure 2:**
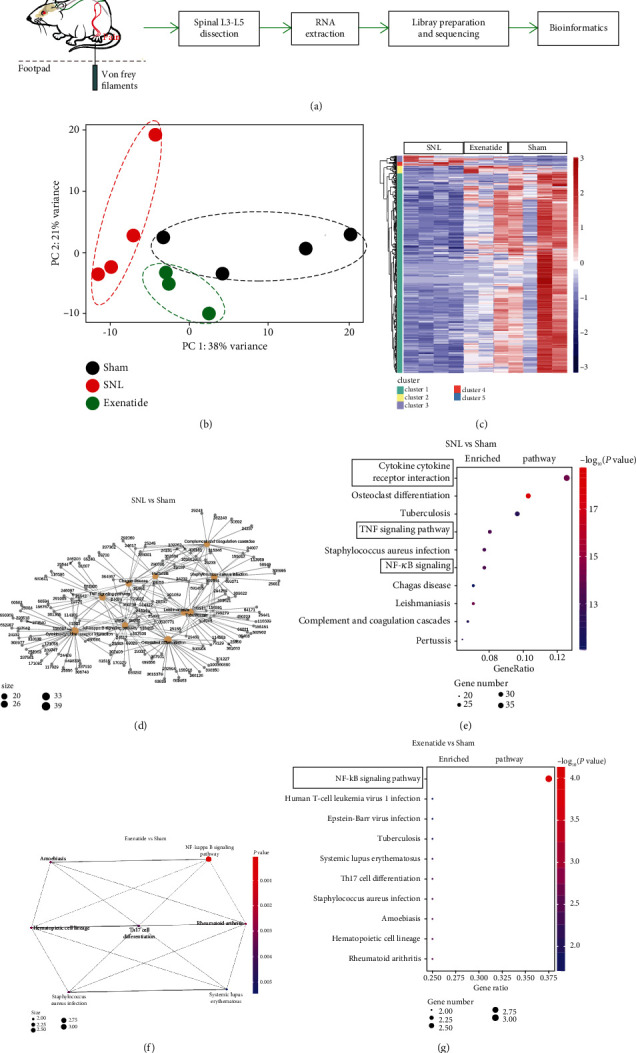
Schematic diagram of the operational process (a). RNA-seq analysis of glucagon-like peptide 1 receptor agonism exenatide induced responses after spinal nerve ligation. Unbiased principal component analysis (b) and heat map (c) of mRNA transcriptome in the spinal ipsilateral dorsal horn. The network of genes (d) and the Kyoto Encyclopedia of Genes and Genomes pathways (e) differentially enriched in the SNL and sham groups. The network of genes (f) and signaling (g) differentially enriched in the exenatide and sham groups (*p* < 0.05). Gene number: number of target genes in each pathway. Rich factor: the ratio of the number of target genes divided by the number of all the genes in each pathway.

To examine gene expression profiles in neuropathic pain via the regulation of microglial GLP-1R signaling, RNA sequencing (RNA-seq) analysis of GLP-1R signaling in neuropathy was performed using an Illumina NovaSeq 6000 PE150 platform and analyzed using R packages (Figure 2(a)). A multidimensional scaling analysis (principal component analysis, PCA) revealed a clear separation between the sham group and the SNL group, and the exenatide-treated group showed a tendency towards the sham group (Figure 2(b)). We then analyzed differentially expressed genes across the three groups by comparison. Of the 24416 detected mRNA genes profiled, 591 (2.42%) were involved in the spinal dorsal horn following SNL (Figure 2(c)). Nerve injury induced a large scale of dysfunctions of genes; more importantly, exenatide induced a recovery towards the sham group, which was consistent with the distribution of PCA. To investigate the potential cell signaling pathways associated with differential gene expression in GLP-1R signaling, pathway interactions were constructed (Figure 2(d)). The Kyoto Encyclopedia of Genes and Genomes (KEGG) analyses revealed that nerve injury triggered more than 20 signaling genes involved, including NF-*κ*B signaling, TNF-*α*, Toll-like receptor, and cytokine-cytokine receptor interaction, and this signaling was associated with inflammation and the inflammation-induced sensitization of synaptic transmission (Figure 2(e)). The NF-*κ*B pathway was associated enriched in the KEGG when the intrathecal injection of exenatide through the large scales of RNA expression was compared with the sham group (Figures 2(f) and 2(g)).

### 3.3. WGCNA Identifies Critical Modules that Correlate with the GLP-1R Pathway in Neuropathic Pain

**Figure 3:**
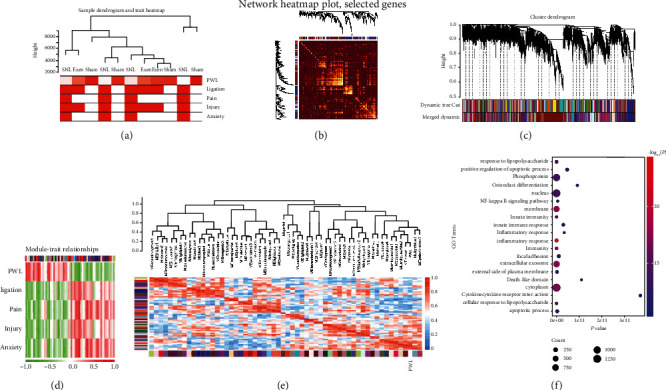
Weighted gene coexpression network analysis of microglial glucagon-like peptide 1 receptor signaling, hierarchical clustering dendrogram of the samples based on paw withdrawal latency, ligation, injury, pain, and anxiety (a), topological overlap matrix (TOM) among identified genes (b), average linkage hierarchical clustering dendrogram of the genes. Input was the TOM based on dissimilarity. Modules, designated by color code, are the branches of the clustering tree (c), correlation between module character and pathological traits. Each color represents the correlated degree. The values are presented as “Pearson *r* (*p* value),” and color-coded by the degree of the correlation (red = positive correlation; green = negative correlation) (d), unsupervised hierarchical clustering heat map, color-coded by the degree of the correlation (red = positive correlation; blue = negative correlation) (e), pathway analysis using the Kyoto Encyclopedia of Genes and Genomes showing the top pathways enriched in the gene sets of the brown, distance between nodes is determined by *p* value and gene counts (f).

To explore the relationship between critical gene models of GLP-1R clusters and modules in neuropathic pain, WGCNA was carried out. The connections of the phenotype and variations in gene expression were then tested to summarize the associated modules. The neuropathic pain model was characterized by ligation, nerve injury, pain, anxiety, and paw withdrawal latency. Based on these phenotypes, highly correlated gene expression was calculated using the R package of WGCNA, and a sample dendrogram associated with traits was established (Figure 3(a)). The topological overlap matrix (TOM) plot of the weighted network shows high similarity in network construction (Figure 3(b)). Highly similar modules were separated by clustering and grouped together (Figure 3(c)). Our data identified 58 modules with one large module and several smaller modules in neuropathic pain by WGCNA (Figures 3(a)–3(c)). Of the 58 modules correlated with behavioural tests and operation in neuropathic pain, the relationships were qualified by R packages, which thoroughly concluded the interactions (Figure 3(d)). The correlation of each module is shown in a heatmap (Figure 3(e)). The largest gene modules (brown) strongly correlated with paw withdrawal latency indicated that paw withdrawal latency was significantly associated with gene expression in neuropathic pain. Based on the most correlated module with paw withdrawal latency, the brown module was further illustrated. We found the brown module enriched for inflammatory responses including response to lipopolysaccharide, NF-*κ*B signaling pathway, innate immunity, innate immune response, inflammatory responses, and cytokine-cytokine receptor interaction by the KEGG analysis (Figure 3(f)).

### 3.4. Microglial GLP-1R Signaling Is Associated with the Excitability of Neurons in SG through MOR

**Figure 4:**
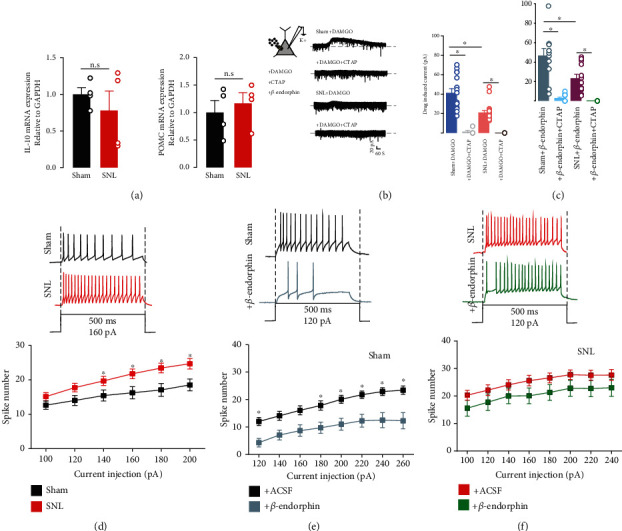
Expression of glucagon-like peptide 1 receptor/IL-10/*β*-endorphin/*μ*-opioid receptor (MOR) in the ipsilateral side. IL-10 and *β*-endorphin mRNA (a), MOR agonist DAMGO (b), and *β*-endorphin (c) induced outward currents, excitability (d), and inhibitory effect of *β*-endorphin on the sham (e) and SNL (f) groups. Rats were induced with neuropathy by the ligation of spinal nerves. Neurons were obtained from the lamina II spinal dorsal horn neurons. The data are presented as the means ± SEM (*n* = 5-6 animals). ^∗^*p* < 0.05, by one-way ANOVA followed by the Tukey post hoc test.

Neuropathic pain arises from the dysfunction of neuronal circuits of noxious and nociceptive information; however, the direct evidence of neuroplasticity in the GLP-1R pathway has been debated. A previous study from our laboratory revealed that the microglial GLP-1R agonist exenatide exerted antiallodynia by stimulating the twofold expression of IL-10/*β*-endorphin. To our knowledge, *β*-endorphin binds to *μ*-receptor which showed inhibitory effects on neurons and was associated with neural plasticity. However, this pathway could not be distinguished from the RNA-seq data. To highlight the direct association between GLP-1R/IL-10/*β*-endorphin signaling and neural plasticity, neuronal excitability was assayed by application of MOR ligands, *β*-endorphin or DAMGO. Indeed, the mRNA expression level of IL-10 and *β*-endorphin was not significantly changed in the SNL group, as compared with the sham group (Figure 4(a)), while the functions of MOR are under debate. Activation of MOR induced an outward current when bath application of DAMGO (1 *μ*mol) and *β*-endorphin (1 *μ*mol) and pretreatment with CTAP (1 *μ*mol) completely reversed these currents in both the sham and SNL groups. Moreover, the currents were more obviously inhibited in the SNL group compared with the sham group (Figures 4(b) and 4(c)). MOR was associated with the excitability of neurons in SG. Current clamp recordings of spikes were generated by direct intracellular current injections and used to assess the excitability of neurons. Increased spikes were generated in the SNL group compared to the sham group (Figure 4(d)). In the sham group, current injection induced declined spikes when using the bath application of *β*-endorphin, while this inhibitory effect was more likely abolished in the SNL group, indicating that activation of MOR is more likely to inhibit the excitability of the sham group and downregulation of MOR-coupled signaling due to nerve injury (Figures 4(e) and 4(f)).

## 4. Discussion

Neuropathic pain is characterized by abnormal neural circuits [17, 18]. Crosstalk between the microglia and neurons has gained more attention in pain. Manipulation of the microglial functions has shown great potential in antiallodynia. Our data supported that activation of spinal microglial GLP-1R signaling reversed nerve injury followed by comprehensive transcriptional changes. Specifically, inflammatory responses were identified as the highest-correlated signaling pathway with paw withdrawal latency.

Neuropathic pain is a widespread disease worldwide, and it is important to discover and illustrate new targets and pathways. Our previous study revealed that microglial GLP-1R agonism inhibited various pain states through autocrine IL-10/STAT3 and cAMP/PKA/p38*β*/CREB signaling in primary microglia cultures [10, 19]. At the early stage, microglial-derived inflammatory factors contribute to abnormal neural circuits in pain. Recently, we demonstrated that microglia are promising targets for antinociception and neuroprotection. A previous study demonstrated that the GLP-1R agonist exenatide suppressed mechanical allodynia in a dosage- and time-dependent manner in vivo, and a primary culture study also demonstrated that exenatide stimulated *β*-endorphin expression in vitro.

To study GLP-1R signaling in a more comprehensive way, the RNA-seq was performed. Compared with the previous study, bioinformaticians could provide independently and reliably transcriptional information. The large-scale mRNA screening by next-generation sequencing identified 591 genes involved in pain, and our results highlighted that intrathecal delivery of exenatide rescued the abnormal gene expression. However, nerve injury triggered the activation of microglia with upregulation of IL-6, IL-1*β*, and TNF-*α*, which modulate neuronal receptors and channels, resulting in hypersensitivity. As shown in Figure 2, the KEGG analysis showed that inflammatory signaling of TNF-*α*, Toll-like receptor, and cytokine-cytokine receptor interaction were enriched. However, intrathecal administration of exenatide shifted towards the sham group; the KEGG analysis found that NF-*κ*B signaling is mainly enriched and also demonstrated that GLP-1R signaling completely inhibited pain through a large scale of gene expression. Our results highlight the broad significance of GLP-1R signaling in the regulation of pain states.

WGCNA uncovered 58 modules in our data in accordance with the pathological traits in our operation. In our rodent model of neuropathic pain, the SNL model was operated by ligation of spinal L5 and L6, and after 14 days, the microglial-derived inflammatory response accelerated nerve injury and developed to pain states [20, 21]. In human and animal studies, many studies have found that patients with pain are more likely to develop anxiety-like behaviours [22–26]. Periphery nerve injury sensitized spinally mediated transmission of messengers of pain to a higher brain control center, including lateral parabrachial area, caudal ventrolateral medulla, and periaqueductal grey matter. Considering the comorbidity of anxiety with pain, anxiety was selected as one element in WGCNA [27]. For further analysis, pathological traits of ligation, nerve injury, pain, anxiety, and paw withdrawal latency were chosen. More importantly, paw withdrawal latency is an objective assessment compared with pathological traits of ligation, nerve injury, pain, and anxiety [28, 29]. We found 58 modules in exenatide-induced antiallodynia by WGCNA. Activation of microglia and astrocytes accelerate the release of inflammatory factors, which in turn is considered a key element in neuropathic pain [30, 31]. All the modules were associated with parameters of behavioural hypersensitivity which may further support the promising mechanism of inhibition of inflammation in pain and the strategy employed in drug development. Interestingly, a previous study on RNA-seq found that nerve injury was characterized by inflammatory responses and highlighted response to lipopolysaccharide, inflammatory response, immune response, and cellular response to lipopolysaccharide signaling [32]. The application of the GLP-1R agonist exenatide inhibited hypersensitivity; meanwhile, response to lipopolysaccharide, inflammatory response, immune response, and cellular response to lipopolysaccharide pathway were the most correlated signaling pathways in exenatide-induced neuroprotection. This finding revealed that anti-inflammatory therapy could be an effective approach for neuropathic pain treatment.

Nerve injury is associated with glial inflammation, leading to maladaptive response. Inflammatory factors (TNF-*α* and IL-1*β*) contribute to neuronal dysfunction associated with ion channel activity, subunits of channels, and phosphorylation of kinases which in turn may regulate the excitability of SG neurons, consistent with RNA-seq data of inflammatory reaction [33, 34]. Many studies have highlighted that microglia is crucial for maintaining synaptic plasticity in neuropathic pain [35, 36] and demonstrated that the modulation of microglial receptors or functions also inhibits pain states [36–38]. Interestingly, our previous study verified that microglial GLP-1R agonist exenatide suppressed various pain states, and pretreatment of IL-10 antiserum and *β*-endorphin antiserum completely reversed the inhibition of allodynia [10, 11]. Another previous study from our laboratory found that microglial autocrine IL-10 suppressed hypersensitivity in neuropathic pain and was separated from inflammatory responses [19]. Intrathecal administration of IL-10 stimulates POMC expression and binding to presynaptic neurons to mitigate nerve injury-induced hypersensitivity in the spinal dorsal horn of the SG [19, 39]. IL-10 suppressed LPS-induced TNF-*α*, IL-1*β*, or IL-6 expression without affecting mechanical allodynia during the inhibition of MOR. In addition, siRNA/Bcl3 and Socs3, responsible for IL-10 suppressing the anti-inflammatory cytokine expression, totally inhibited TNF-*α*, IL-1*β*, and IL-6 expression but not mechanical allodynia [19]. Intrathecal administration of spinal microglial GLP-1R agonist suppressed hypersensitivity, and WGCNA also indicated that exenatide totally abolished inflammatory responses, which further supports our previous research on the separation between pain and inflammation. Moreover, microglial GLP-1R signaling interrupts abnormal nociceptive information transmission of glutamatergic transmission mediated by autocrine IL-10 and *β*-endorphin expression, which in turn abolished nociceptive circuits from peripheral receptors to the brain mediated by the spinal cord mediated by MOR [10, 13, 19]. Those data supported that microglial GLP-1R signaling exhibited neuroprotection through binding to neuronal expressed inhibitory MOR.

Intrinsic plasticity refers to changes in the excitability of neurons during pain. Changes in neuronal excitability are a major feature of hypersensitivity and are still disputed when activating GLP-1R signaling. As we know, MOR was the typical opioid in neuropathic pain and is associated with the excitability of neurons. In order to illustrate the role of GLP-1R/IL-10/*β*-endorphin signaling modulating neuronal excitability, MOR agonism *β*-endorphin and DAMGO were applied. Bath application of MOR agonist *β*-endorphin and DAMGO stimulated outward currents in the SG neurons [39], and this inhibitory function was damaged after nerve injury, consistent with our study, showing that the excitability of neurons in the sham group was more sensitive to *β*-endorphin compared with the SNL group. In that line, MOR intervention yielded several mechanisms, including balance of the neural circuits, mainly GABAergic and glycinergic neurons in the spinal cord, and reduction of the inhibition of secondary GABAergic interneurons [40].

**Figure 5:**
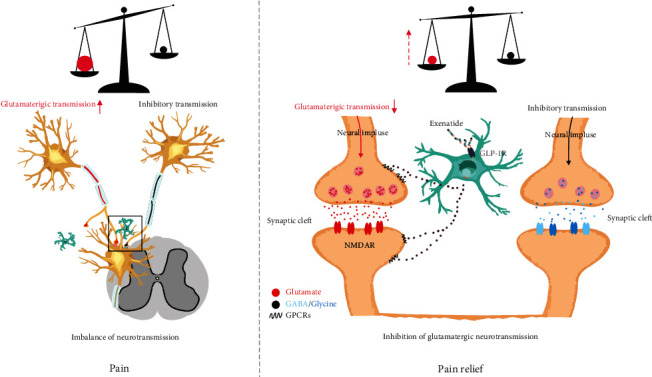
Schematic diagram showing that microglial glucagon-like peptide 1 receptor agonist exenatide prevented peripheral spinal nerve injury-induced mechanical allodynia and glutamatergic transmission and rescued 591 gene expressions associated with a spinal microglial-mediated mechanism.

In summary, behavioural tests and whole-cell recording of the sham, SNL, and exenatide groups indicated strong connections between GLP-1R and neuronal excitability, mechanical allodynia, hypersensitivity, and neuropathic pain. It is worth noting that the intrathecal delivery of exenatide prevented nerve injury-induced damage. WGCNA highlighted a strong correlation between gene expression and functional changes including ligation, hypersensitivity, and anxiety, which established the microglial GLP-1R pathway in pain management, and highlighted key elements of pain-associated targets of spinal microglial GLP-1R in future pharmaceutical development, as shown in Figure 5.

## Data Availability

All data, models, or figures generated or used during the study are available from the corresponding authors.

## Abbreviations

SNL: Spinal nerve ligation
GLP-1: Glucagon-like peptide-1
ACSF: Artificial cerebrospinal fluid
mEPSC: Miniature excitatory postsynaptic current
SG: Substantia gelatinosa
DESeq2: Differential gene expression analysis based on the negative binomial distribution
WGCNA: Weighted correlation network analysis
KEGG: Kyoto Encyclopedia of Genes and Genomes.
